# Healthcare Access and Utilization Among Older U.S. Immigrants: A Population-Based Analysis

**DOI:** 10.1093/geroni/igaf122.3599

**Published:** 2025-12-31

**Authors:** Irtiza Faruque, Aastha Dharia, Mohammad Usama Toseef

**Affiliations:** Oakland University William Beaumont School of Medicine, Auburn Hills, Michigan, United States; Oakland University William Beaumont School of Medicine, Rochester Hills, Michigan, United States; Corewell Health, Farmington Hills, Michigan, United States

## Abstract

Immigrants comprise a growing segment of the aging U.S. population, with those aged 65 and older now representing approximately 14% of all older adults. Despite their increasing numbers, older immigrants often encounter unique barriers to healthcare, including linguistic, financial, and systemic challenges. Yet, there remains limited research evaluating how these individuals perceive access to and quality of care. Using 2018–2022 data from the Medical Expenditure Panel Survey (MEPS), we conducted a retrospective analysis of self-reported healthcare access among adults aged 65 and older. Immigration status was categorized as U.S.-born, long-term immigrants (≥15 years in the U.S.), and recent immigrants (< 15 years in the U.S.). We examined key aspects of usual source of care (USC), including whether respondents reported having a USC, access to after-hours or weekend care, delays in receiving care or filling prescriptions due to cost, and whether providers communicated in the respondent’s preferred language. Survey-weighted logistic regression models revealed that both long-term and recent immigrants were significantly less likely to report having a USC compared to U.S.-born individuals (OR = 0.7 and OR = 0.4, respectively; p = 0.004). Additionally, recent immigrants were more likely to report delayed medical care due to cost (OR = 2.2, p = 0.029). These findings contribute to growing evidence on how immigration status and length of U.S. residency shape access to care among older adults. By identifying disparities rooted in both migration history and broader social determinants, this study informs targeted strategies to promote health equity in aging populations.

